# Description and scanning electron microscopic observation of a new species of the genus *Polycopetta* (Crustacea, Ostracoda, Cladocopina) from an interstitial habitat in Japan

**DOI:** 10.3897/zookeys.294.4846

**Published:** 2013-04-22

**Authors:** Hayato Tanaka, Akira Tsukagoshi

**Affiliations:** 1 Institute of Geoscience, Faculty of Science, Shizuoka University, Oya 836, Suruga-ku, Shizuoka City, Shizuoka Prefecture, 422-8529, Japan

**Keywords:** Endopodite of fifth limb, long spermatozoa, male antennula, pore systems, *Polycopetta*

## Abstract

A new species of the genus *Polycopetta* Chavtur, 1981, *Polycopetta quadrispinata* **sp. n.** is described from the interstitial environment of Mihomasaki Beach in Japan. These observations showed some morphological peculiarities of *Polycopetta quadrispinata* sp. n. compared with its congeners; *Polycopetta monneroni* Chavtur, 1979, *Polycopetta curva* Chavtur, 1979, *Polycopetta bransfieldensis* (Hartmann, 1987), and *Polycopetta pax* Kornicker and Harrison-Nelson, 2005. Three characteristics are described for the first time: (1) a seta with serrated tip on the male antennula, (2) the endopodite of the fifth limb consisting of two podomeres, (3) the long spermatozoa in the male posterior body. More detailed observations of the type species are needed in order to update the generic diagnosis.

## Introduction

The genus *Polycopetta*, belonging to the Suborder Cladocopina, was originally proposed by Chavtur (1979), but the name of this genus could not be used because the type species had not been designated. Subsequently, Chavtur (1981) assigned the type species and gave a diagnosis for the genus, after which *Polycopetta* Chavtur, 1981 was accepted as a valid name. The generic diagnosis of *Polycopetta* based on Chavtur (1981) is as follows: Carapace oval and concave anteriorly; frontal organ consists of one seta split at distal half; third podomere of male antennula with two ventral setae; male antennal endopodite bearing dorsal outgrowth and hook-like protrusion, and terminal podomere in both sexes with ventral protuberance; basis of fifth limb with one internal and three external setae, and exopodite with four terminal setae; outgrowth between the furcal lamellae rounded and armed with spines; male left furcal lamella with six claws.

Thus far four species have been described in this genus: the type species *Polycopetta monneroni* Chavtur, 1979, *Polycopetta curva* Chavtur, 1979, *Polycopetta bransfieldensis* (Hartmann, 1987) and *Polycopetta pax* Kornicker & Harrison-Nelson, 2005. Three species are found in the sediment of the seafloor at depths of 60 to 265m, except for *Polycopetta pax*. This species has been reported from a *Riftia pachyptila* (giant tube worm) aggregation at a depth of 2500m.

During the faunal survey along the Pacific coast in Japan, a species of *Polycopetta* was found in interstitial habitats for the first time. In the present paper, the authors describe this new species, including observations of the detailed structure of the carapace and appendages, obtained by using a scanning electron microscope (SEM).

## Materials and methods

**Figure 1.**
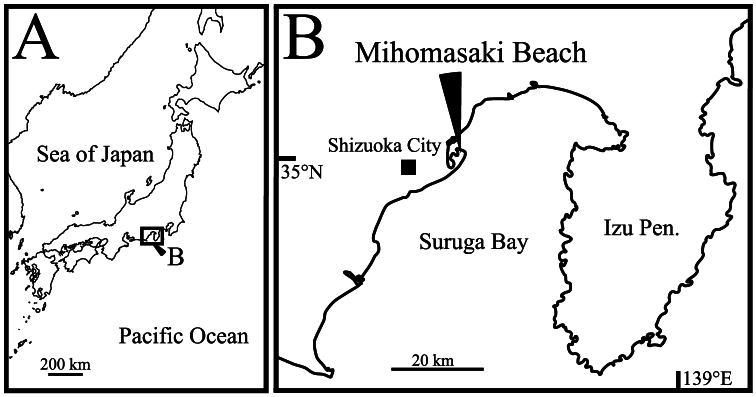
Sampling site. **A** map of Japan **B** type locality of *Polycopetta quadrispinata* sp. n.

Sand material was collected from the Mihomasaki beach, Shizuoka City, Shizuoka Prefecture, Japan (Fig. 1) at 40 cm below the shoreline sand surface, at low tide. The samples were washed five times in a bucket with fresh water, and the top layer of water was strained through nets of 40 μm mesh size. The living specimens were picked out from the remaining deposits under a stereo-binocular microscope (SZH 10, OLYMPUS). The observed specimens were fixed in 8% formalin with neutral buffer (hexamethylenetetramine), and preserved in 80% ethanol at room temperature. The soft parts and valves were dissected with fine needles and mounted in Neo-Shigaral (Shiga Konchu Fukyusha, Tokyo, Japan), or glycerine, on glass slides under a stereo-binocular microscope, and then observed and sketched using a transmitted-light binocular microscope (BX 50, OLYMPUS) with a differential interference contrast system and a camera Lucida. The valves and soft parts, treated with the t-butyl alcohol freeze-drying method, were also coated with osmium and observed by SEM (JSM-5600LV, JEOL).

The type series was deposited in the collection of the Shizuoka University Museum, identified by registration numbers with prefix SUM-CO.

## Taxonomy

### Order Halocyprida Dana, 1853 Suborder Cladocopina Sars, 1866 Family Polycopidae Sars, 1866 Genus *Polycopetta* Chavtur, 1981

#### Polycopetta quadrispinata sp. n.

urn:lsid:zoobank.org:act:2247D429-CEB0-4BA8-BD51-D9FF89F995FD

http://species-id.net/wiki/Polycopetta_quadrispinata

Figs 2 13

##### Type series.

Holotype: adult male (SUM-CO-2093), right valve length 357 μm, height 301 μm, left valve length 358 μm, height 288 μm, soft parts mounted on a slide and valves preserved in a cardboard cell slide, Paratypes: 10 adult males (SUM-CO-2094–2103) and 11 adult females (SUM-CO-2104–2114). All specimens were collected on 24 June 2009.

##### Type locality.

The holotype specimen was collected from Mihomasaki beach, Shizuoka City, Shizuoka Prefecture, along the Pacific coast of central Japan, 35°01'13"N, 138°31'20"E (Fig. 1B); in an interstitial environment at 40 cm below the shoreline sand surface. The substrate consisted mainly of clastic very coarse sand (median grain size is about 1.5 mm).

##### Diagnosis.

Carapace oval and anteriorly concave in lateral view. Carapace peripheral surface covered with shallow pits, except on posterodorsal area, and with scale-like sculptures in anterior area. Anterior end of both valves with one conspicuous spine. Posteroventral margin of right valve with four conspicuous spines in both sexes. In each valve, 73 simple pores, 23 pore systems involving a circular depression and two contiguous pore systems. Male second podomere of the antennula with one ventral seta, third podomere with one ventral seta with serrated tip. Spermatozoa length approximately 750 μm.

##### Description of adult male.

**Figure 2.**
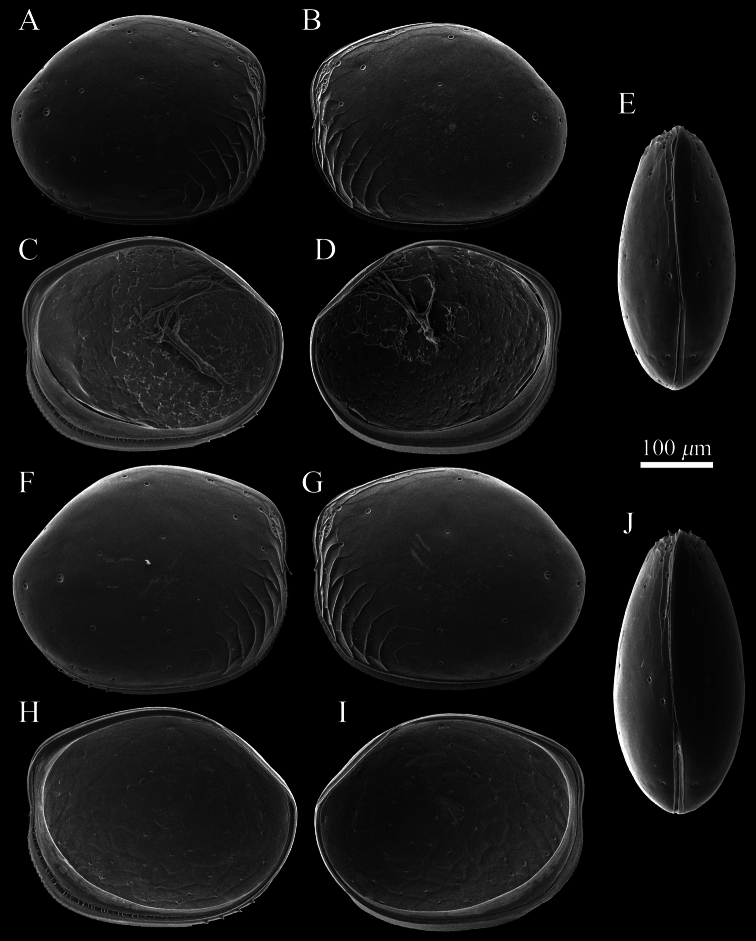
SEM images of *Polycopetta quadrispinata* sp. n. valves. **A** and **B** male paratype (SUM-CO-2095) **C** and **D** male, paratype (SUM-CO-2096) **E** male, paratype (SUM-CO-2097) **F** female, paratype (SUM-CO-2105) **G** female paratype (SUM-CO-2106) **H** and **I** female, paratype (SUM-CO-2107) **J** female, paratype (SUM-CO-2108). **A** right external lateral view **B** left external lateral view **C** right internal lateral view **D** left internal lateral view **E** dorsal view **F** right external lateral view **G** left external lateral view **H** right internal lateral view **I** left internal lateral **J** dorsal view.

**Figure 3.**
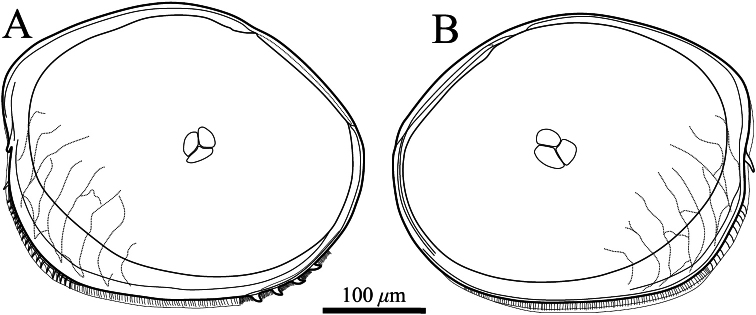
Valves of *Polycopetta quadrispinata* sp. n. Male, holotype (SUM-CO-2093). **A** right internal view **B** left internal view.

**Figure 4.**
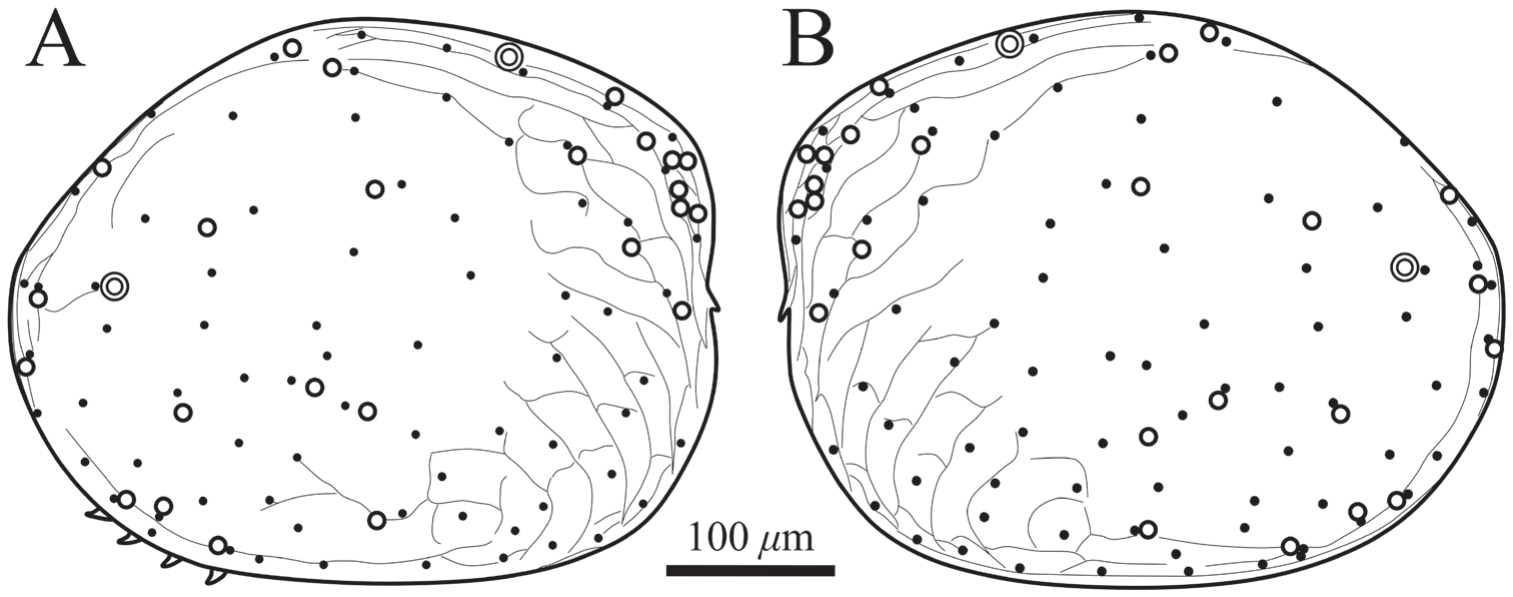
Distribution of pore systems and surface ornamentation of *Polycopetta quadrispinata* sp. n. drawn from an SEM image. Male, paratype (SUM-CO-2095). **A** right external view **B** left external view. Solid circle, open circle and double circle indicate the positions of simple pores, pore systems involving a circular depression and contiguous pore systems, respectively.

**Figure 5.**
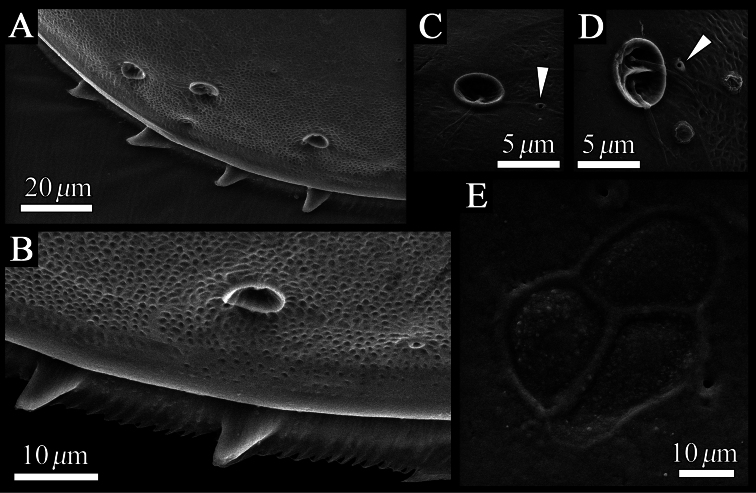
SEM images of the detailed structure of *Polycopetta quadrispinata* sp. n. valves. **A** male, paratype (SUM-CO-2095) **B–D** male, paratype (SUM-CO-2098) **E** male, paratype (SUM-CO-2094). **A** four spines at posteroventral margin of right valve **B** spines and fringe at posteroventral margin of right valve **C** pore system with circular depression **D** contiguous pore system **E** internal lateral view of adductor muscle scars of right valve. Arrowheads indicate simple pores.

**Figure 6.**
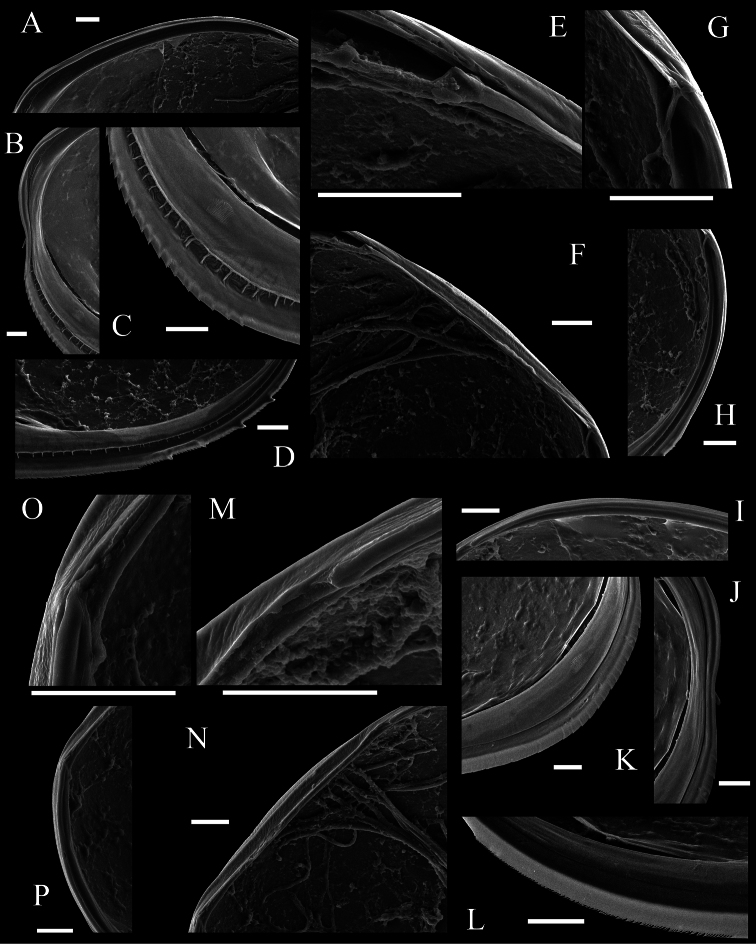
SEM images of internal view of *Polycopetta quadrispinata* sp. n. valves. Male, paratype (SUM-CO-2096). **A–H** right valve **I–P** left valve **A** anterodorsal bar and groove **B** anterior area of marginal infold **C** anteroventral area of marginal infold **D** posteroventral area of marginal infold **E** anterior socket of hinge structure **F** median bar of hinge structure **G** posterior socket of hinge structure **H** posteroventral groove **I** anterodorsal bar **J** anterior area of marginal infold **K** anteroventral area of marginal infold **L** posteroventral area of marginal infold **M** anterior knob of hinge structure **N** median bar of hinge structure **O** posterior knob of hinge structure **P** posterior bar. Scale bars indicate 20 μm.

Carapace (Figs 2A–E, 3–6). Carapace oval and anteriorly concave in lateral view. Yellowish white colour in living individuals. Carapace periphery surface covered with shallow pits except on posterodorsal area, and with scale-like sculptures on anterior area (Figs 2A, B, 3, 4). Anterior end of both valves with one conspicuous spine (Figs 2A, B, 3, 4). Mid-anterior to posteroventral margin covered with serration and fringe (Figs 2A, B, 3). Posteroventral margin of right valve with four conspicuous spines and fringe (Fig. 5A, B) and of left valve with fine serrations (Figs 2B, 3B). In each valve, 73 simple pores (Figs 4, 5C, D), 23 pore systems involving a circular depression with bifurcated seta (Figs 4, 5C) and two contiguous double pore systems with bifurcated seta (Figs 4, 5D). Adductor muscle scar consisting of three closely spaced scars (Fig. 5E). Marginal infold of each valve developed along anterior to posteroventral margins (Figs 2C, D, 6B, C, J–L). Along hinge margin of right valve: anterodorsal bar and groove (Fig. 6A), anterior socket (Fig. 6E), median bar (Fig. 6F), posterior socket (Fig. 6G), and posteroventral groove (Fig. 6D, H). Along hinge margin of left valve: anterodorsal bar (Fig. 6I), anterior knob (Fig. 6M), median bar (Fig. 6N), posterior knob (Fig. 6O), and posterior bar (Fig. 6P).

**Figure 7.**
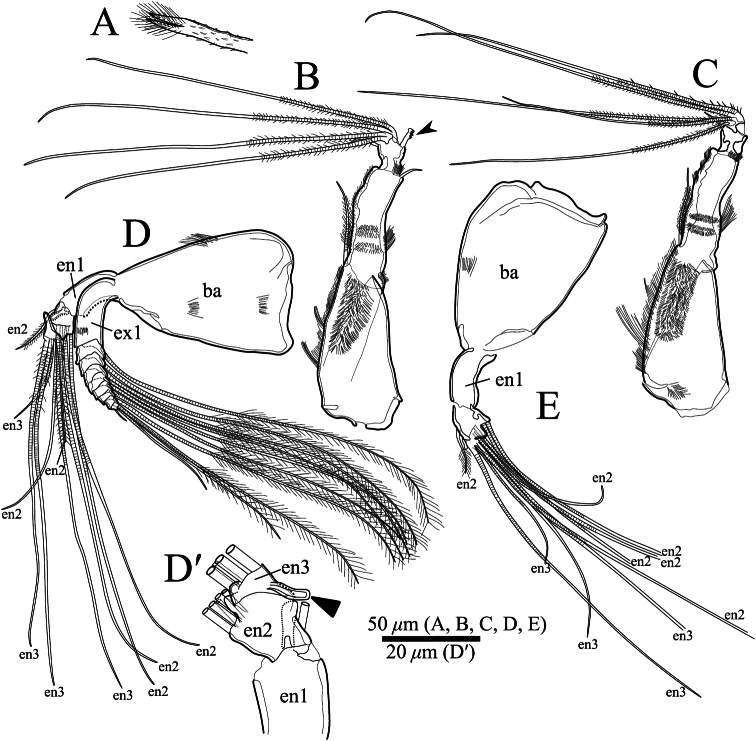
*Polycopetta quadrispinata* sp. n. **A**, **B** and **D** male, holotype (SUM-CO-2093) **C** and **E** female, paratype (SUM-CO-2104). **A** frontal organ **B** male antennula **C** female antennula **D** male antenna **D**′ endopodite of male antenna without all setae **E** female antenna except the exopodite. Abbreviations: **ba** basis **en** endopodite **ex** exopodite.

**Figure 8.**
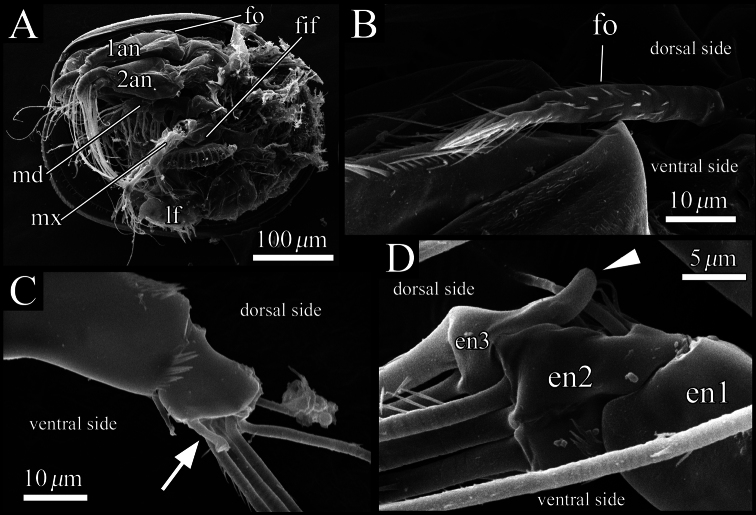
SEM images of male soft parts of *Polycopetta quadrispinata* sp. n. **A** left lateral view of whole body with left valve removed **B** left lateral view of frontal organ **C** right lateral view of distal area of antennula **D** left lateral view of antennal endopodite. Arrow indicates a seta with serrations at ventrodistal end. Arrowhead indicates a dorsal outgrowth of male antennal endopodite. Abbreviations: **1an** antennula **2an** antenna **en** endopodite **fif** fifth limb **fo** frontal organ **lf** left furcal lamella **md** mandibula **mx** maxillula.

Frontal organ (Figs 7A, 8B). Spinous seta divided at mid-length. Distal half with long and proximal half with short setae, respectively (Fig. 8B).

Antennula (Figs 7B, 8C). Uniramous, four articulated podomeres. First podomere rectangular in shape and tapering distally, with setulae on dorsal margin, lateral surface and at ventrodistal end, respectively. Second podomere about four-fifths as long as first podomere, with one annulated setulous seta at dorsoproximal end, one simple seta on ventrodistal end (Figs 7B, 8C), and setulae on dorsal margin, lateral surface, ventral middle margin and at ventrodistal end, respectively. Third podomere about one-fifth as long as first podomere, with one short simple seta at dorsodistal end and one seta with serrations at ventrodistal end (Figs 7B, 8C). Fourth podomere small, with four long setulous annulated setae.

Antenna (Figs 7D, D', 8D). Typically biramous, with exopodite and endopodite consisting of nine and three podomeres, respectively. Basis triangular and tapering distally. Exopodite: first podomere about one–third as long as basis; podomere lengths decreasing in size from second to eighth, each podomere with one long plumose annulated seta, respectively; ninth (distal-most) podomere very small, with one long annulated, one medium annulated and one short bare setae at distal end. Endopodite (Figs 7D, D', 8D): first podomere about two-thirds as long as first podomere of exopodite; second podomere half as long as first podomere, with one setulous seta along dorsal margin, one clavate process at proximal middle end (Fig. 7D') and five setae at distal end consisting of three long annulated, one medium annulated and one short annulated setulous. Third podomere one-fifth as long as first podomere, with one dorsal outgrowth (Figs 7D', 8D), and two long spinous annulated, one long annulated and one short setulous annulated setae at distal end.

Upper lip (Fig. 10A). Semicircular in lateral view, with fine setae on surface (Fig. 13A).

Mandibula (Fig. 10B). Coxal endite with four teeth. Basis with four plumose annulated setae on ventral margin, and one plumose annulated seta at mid-lateral surface. Exopodite pear-shaped, distal end jagged, with thin setae, and one simple seta. Endopodite consisting of two podomeres. First podomere with three annulated plumose setae on ventral margin and two annulated long setulous setae at dorsodistal end. Second podomere very small, bearing two plumose setae at distal end.

Maxillula (Fig. 10C, C', C"). Precoxa (Fig. 10C') with seven annulated plumose setae and one stout setulous seta on ventral side. Coxa (Fig. 10C") with two short and two medium plumose setae on lateral surface near ventroproximal margin, two short and two medium plumose setae on lateral surface of ventral middle margin. Basis rectangular, dorsally-convex in lateral view, with one medium and one long plumose setae on ventral margin, and setulae along ventral margin. First podomere of endopodite with one long plumose seta at ventrodistal end. Second podomere three-fourths as long as first podomere, with two long and one medium annulated setulous setae on ventrodistal area, one short annulated and one medium setulous annulated seta at dorsodistal end. Third podomere small, with 4 long annulated setulous setae. Exopodite with four tufts along dorsal margin, and nine annulated setae at distal end.

Fifth limb (Figs 10D, 11). Coxa bearing branchial plate (epipodite) with 15 long plumose setae, and four short setulous setae on dorsolateral area. Basis with three setulous and three plumose setae on dorsal and ventral margin, respectively. Endopodite consisting of two podomeres (Fig. 11). First podomere with one plumose seta. Second podomere rectangle, with one plumose seta. Exopodite with four setulous setae.

Furca (Figs 12A, 13B). Furcal claws six and seven on left and right lamella, respectively, with row of setae on dorsal side.

Male copulatory organ and posterior body (Figs 12A, 13B–D). Arising from outer surface of body on left side of terminal trunk segment as long curved copulatory duct. Tuft of stout setae at ventral right side (Fig. 12A, 13C). Posterior body including a lot of very long spermatozoa, approximately 750 μm long (Fig. 12A, 13D).

##### Description of adult female.

Mandibula, maxillula, fifth limbs, and upper lip similar to those of adult male.

Carapace (Fig. 2F–J). Carapace length and height larger than adult males.

Antennula (Fig. 7C). Uniramus, four articulated podomeres. First podomere similar to that of adult male. Second podomere about four-fifths as long as first podomere, with one annulated setulous seta at dorsoproximal end, and setulae on dorsal margin, lateral surface, ventral middle margin and at ventrodistal end, respectively. Third podomere about one-fifth as long as first podomere, with one short simple seta at dorsodistal end. Fourth podomere small, with five long setulous annulated setae.

Antenna (Fig. 7E). Only second and third podomeres of endopodite different from those of adult male. Endopodite consisting of three podomeres. Second podomere half as long as first podomere, with one setulous seta along dorsal margin and five annulated setae at distal end. Third podomere one-fifth as long as first podomere with four annulated setae at distal end.

Furca (Fig. 12B). Each lamella with seven claws.

##### Dimensions.

See Table 1.

**Table 1.** Dimensions of valves of *Polycopetta quadrispinata* sp. n. from type locality.<br/>

			**Length (μm)**				**Height (μm)**	
		**Mean**	**Observed range**	**N**		**Mean**	**Observed range**	**N**
Male	Right valve	357	353–361	10		296	292–301	10
	Left valve	356	353–364	10		290	288–294	10
Female	Right valve	380	372–388	9		312	306–322	9
	Left valve	381	373–389	9		308	302–316	9

**Figure 9.**
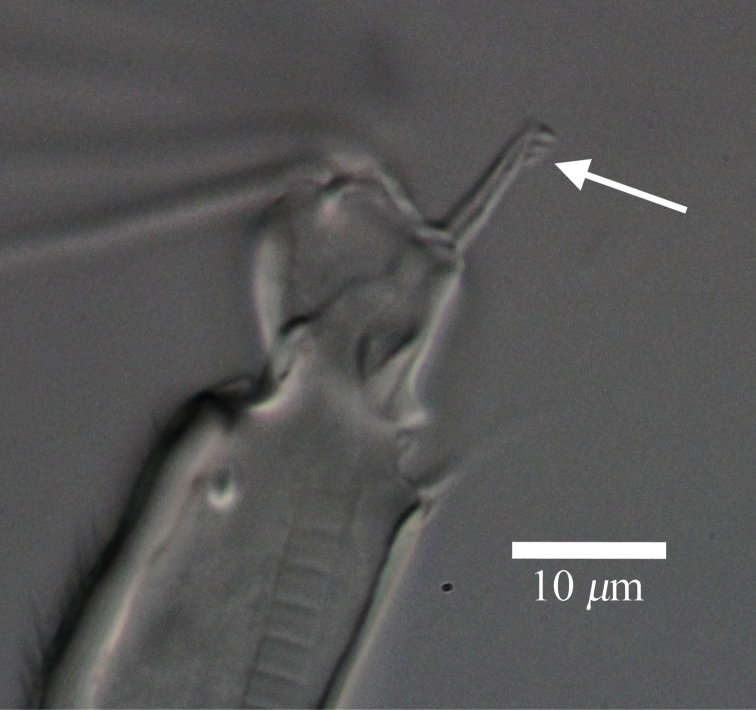
Light micrograph of male antennula of *Polycopetta quadrispinata* sp. n. Arrow indicates seta with serrations at ventrodistal end.

**Figure 10.**
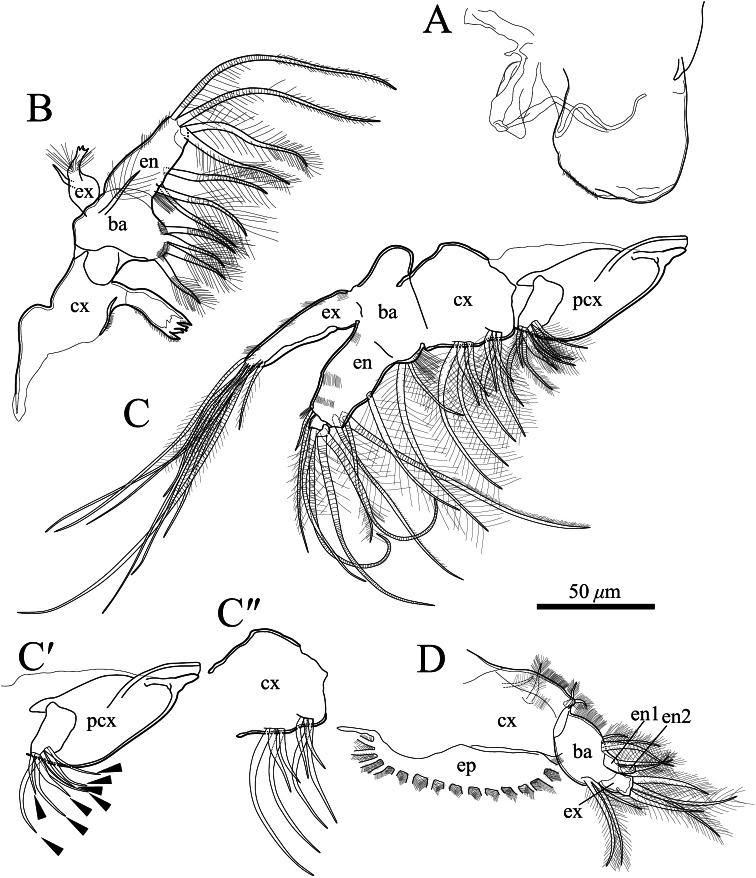
*Polycopetta quadrispinata* sp. n. **A** and **D** male, paratype (SUM-CO-2094) **B** and **C** male, holotype (SUM-CO-2093). **A** right lateral view of upper lip **B** mandibula **C** maxillula **C**′precoxa of maxillula except setulae on setae **C**" coxa of maxillula except setulae on setae **D** fifth limb. Arrowheads indicate tip of precoxal setae of maxillula. Abbreviations: **ba** basis **cx** coxa **en** endopodite **ex** exopodite **pcx** precoxa.

**Figure 11.**
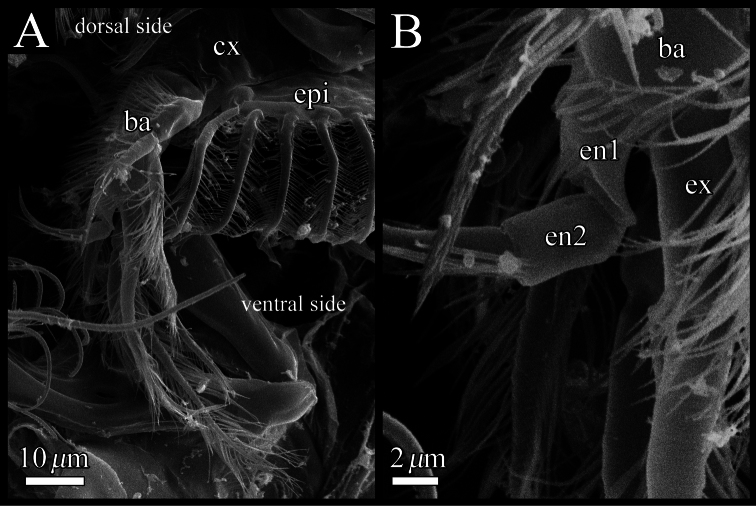
SEM images of male fifth limb of *Polycopetta quadrispinata* sp. n. **A** left lateral view **B** enlarged view of figure **11A**. Abbreviations: **ba** basis **cx** coxa **en** endopodite **ex** exopodite **epi** epipodite.

**Figure 12.**
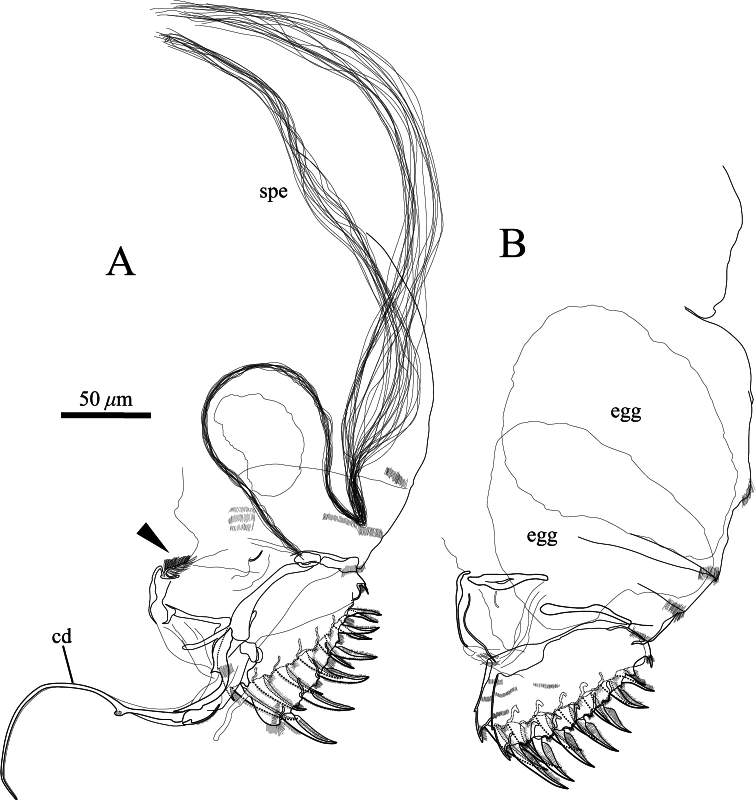
Posterior body of *Polycopetta quadrispinata* sp. n. **A** male, holotype (SUM-CO-2093) **B** female, paratype (SUM-CO-2106). Arrowhead indicates the tuft of stout setae. Abbreviations: **cd** copulatory duct **spe** spermatozoa.

**Figure 13.**
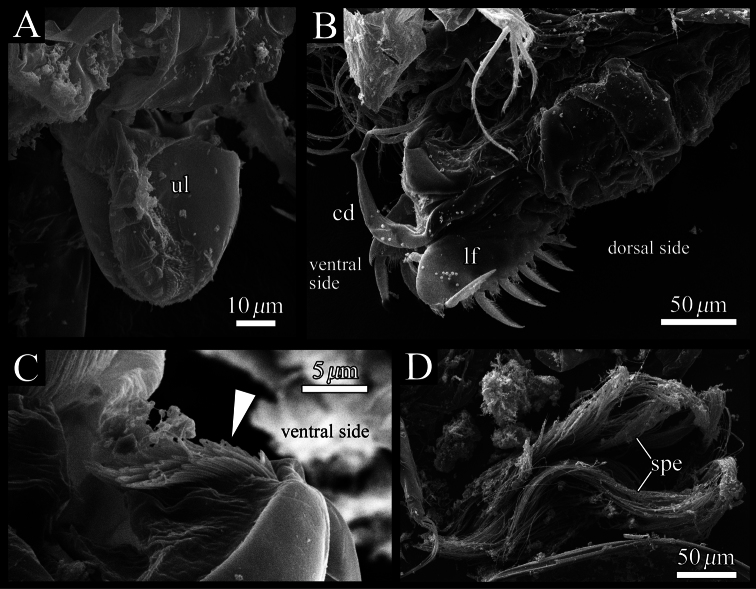
*Polycopetta quadrispinata* sp. n. SEM images of male soft parts of. **A** right lateral view of upper lip **B** left lateral view of posterior body, furcal lamellae and copulatory organ **C** right lateral view of the tuft of stout setae **D** fascicle of spermatozoa. Arrowhead indicates the tuft of stout setae. Abbreviations: **cd** copulatory duct **lf** left furcal lamella **spe** spermatozoa **ul** upper lip.

##### Occurrence.

So far known only from type locality.

**Etymology.** Specific name *quadrispinata*, an adjective derived from the Latin prefix *quadri*- (four) and Latin adjective *spinatus* (spiny), referring to the four spines on the posteroventral margin of the right valve in both sexes.

## Discussion

Existing species of Polycopetta are known from only a few specimens in seafloor sediment and deep sea tube worm aggregations. This study is the first report of a species of *Polycopetta* from the interstitial environment. Because 21 specimens were obtained, the authors could observe the details of their morphologies.

Four species of *Polycopetta* have been described: *Polycopetta monneroni*, *Polycopetta curva*, *Polycopetta bransfieldensis*, *Polycopetta pax*. *Polycopetta quadrispinata* sp. n. and *Polycopetta curva* are similar to each other; i.e. both species have scale-like sculpture on the anterior carapace surface (Fig. 2A, B), one clavate process at proximal middle end of second podomere of male antennal endopodite (Fig. 8D), and four teeth as coxal endites of mandibula (Fig. 10B). They are distinguished by the number of spines at posteroventral margin of right valve, four for *Polycopetta quadrispinata* (Fig. 5A) and one for *Polycopetta curva*, respectively. This new species is distinguishable from each of the other three species by the number of coxal teeth (endites) of the mandibula (two in *Polycopetta monneroni* vs four (Fig. 10B) in *Polycopetta quadrispinata* the carapace surface ornamentation (absent in *Polycopetta bransfieldensis* vs scale-like sculpture and pits (Fig. 2A, B) in *Polycopetta quadrispinata* and the number of adductor muscle scars (six in *Polycopetta pax* vs three (Fig. 5E) in *Polycopetta quadrispinata* This new species also differs from all previously described species by details of the chaetotaxy of the antennula, antenna, maxillula and fifth limb (see Table 2).

**Table 2.** Interspecific morphological comparison of the genus *Polycopetta*. The dashes indicate no information from original descriptions.<br/>

**Character**	***Polycopetta monneroni***	***Polycopetta curva***	***Polycopetta bransfieldensis***	***Polycopetta pax***	***Polycopetta quadrispinata***
Female					
*Carapace*, length (μm)	325–350	450	480–490	540	372–389
Height (μm)	–	350	–	470	302–322
Height/Length (%)	–	78	–	87	79–84
number of spines at posteroventral margin of right valve	–	1	–	6?	4
*Mandible*, coxal endite	2 teeth	4 teeth	–	4 bifurcate teeth	4 teeth
shape of exopodite tip	flat	flat	jagged	flat	jagged
*Maxillula*, seta number on precoxa	7	7	5	5	8
*Fifth limb*, podomere number of endopodite	1	1	1	1	2
seta number of epipodite	–	–	–	12	15
*Furca*, claw number (left-right)	(7-7)	(6?-7?)	(7-7)	(6?-6?)	(7-7)
Male					
*Carapace*, length (μm)	–	450	490	–	353–364
Height (μm)	–	350	–	–	288–301
Height/Length (%)	–	78	–	–	80–85
*Antennula*, seta with serrations at ventrodistal end	absent	absent	–	–	present
*Antenna*, shape of process on 2nd podomere of endopodite	distally tapered hook-like	distally expanded and rounded hook-like	–	–	clavate
*Furca*, claw number (left-right)	(6-7)	(6-7)	–	–	(6-7)

Our observation shows some morphological peculiarities of *Polycopetta quadrispinata* sp. n. when compared with its congeners. First, the third podomere of male antennula bears one seta with serrations at the ventrodistal end (Figs 7B, 8C). This seta has not been identified in the other species. Since this seta is only found in the male, itmust be related to sexual activity; however the function of this seta is unknown at the present time. Second, the endopodite of the fifth limb consists of two podomeres (Fig. 11B). Kornicker and Harrison-Nelson (2005) stated that the podomere number is only one in *Polycopetta pax*. Third, the long spermatozoa (Figs 12A, 13D) are described in *Polycopetta* for the first time. The males have been known for three species (*Polycopetta monneroni*, *Polycopetta curva* and *Polycopetta bransfieldensis*), but there is no information about their spermatozoa. In the family Polycopidae the sperm length of *Eupolycope dispar* (Müller, 1894) and *Polycope cancellea* Hartmann, 1954 have been reported (Hartmann 1955; 1968). The length of the former species is 45 μm (carapace length is 300 μm), the latter is 15 μm (carapace length is 500 μm). The sperm length (750 μm) of the new species is extreme for this family. These characters are likely to be present in other incompletely described species. In future, more detailed observation of all of these species may be needed, in order to update the generic diagnosis.

## Supplementary Material

XML Treatment for Polycopetta quadrispinata
